# Arterial stiffness in individuals with TB and diabetes mellitus using a pulse wave analysis

**DOI:** 10.5588/ijtldopen.26.0355

**Published:** 2026-11-13

**Authors:** M. Kahabuka, Y. Alkabab, A. Chongolo, J.L. Erdal, L.S. Halstad, J.O. Jacobsen, C.S. Grande Mboje, S.K. Heysell, S.G. Mpagama, D.L. Christensen

**Affiliations:** 1 Kibong’oto Infectious Diseases, Sanya Juu, Tanzania;; 2 Medical University of South Carolina, Charleston, SC, USA;; 3 National Institute for Medical Research, Mbeya, Tanzania;; 4 Ludwig-Maximilian’s-University, Munich, Germany;; 5 Department of Public Health, University of Copenhagen, Copenhagen, Denmark;; 6 Division of Infectious Diseases and International Health, University of Virginia, Charlottesville, VA, USA;; 7 The Nelson Mandela African Institution of Science and Technology, Arusha, Tanzania.

**Keywords:** tuberculosis, DM, pulse wave velocity, vascular injury, central augmentation index

Dear Editor,

TB and diabetes mellitus (DM) are co-epidemic conditions disproportionately affecting many low- and middle-income countries (LMICs). As both independently increase cardiovascular disease (CVD), their coexistence represents an increasing public health concern. TB promotes vascular injury through direct effects of *Mycobacterium tuberculosis* on vascular structures, the toxicity of anti-TB drugs, and systemic inflammation, whereas DM accelerates atherosclerosis through chronic hyperglycaemia and broader metabolic dysregulation.^1–3^

Arterial stiffness, measured by carotid-femoral pulse wave velocity (cfPWV), is an independent predictor of CVD morbidity and mortality and is regarded as the gold standard non-invasive surrogate of subclinical atherosclerosis.^4^ Pulse wave analysis (PWA) additionally permits estimation of central haemodynamic parameters, including central augmentation index (AIx, augmentation pressure/pulse pressure × 100 [%]), central systolic blood pressure (cSBP), central diastolic pressure (cDBP), pulse pressure (PP, cSBP − cDBP), and mean arterial pressure (MAP, [SBP + (2 × DBP)]/3), providing a comprehensive and practical risk assessment tool particularly suited to resource-limited settings.^4^ Despite the biological plausibility of a synergistic cardiovascular risk in TB/DM comorbidity, data from sub-Saharan African populations remain scarce, although the region carries a high burden of TB and rapidly growing rates of DM and CVD.^5^ This pilot study sought to describe arterial stiffness and cardiovascular risk profiles among individuals with TB only, DM only, and TB/DM comorbidity in the Kilimanjaro Region of Tanzania.

This cross-sectional sub-study was nested within the ADEPT study in the Kilimanjaro Region.^6^ Participants aged ≥ 35 years with TB only (defined as having a positive sputum test at enrolment in the ADEPT study and having completed anti-TB treatment without evidence of recurrent or new TB infection), DM only (defined as confirmed DM or hyperglycaemia, with HbA1c ≥ 48 mmol/mol [≥6.5%] and either a random blood glucose ≥ 11 mmol/L or fasting blood glucose ≥ 7.0 mmol/L, managed with medication and/or dietary therapy), or TB/DM comorbidity were re-invited to undergo vascular assessment. PWA was performed using a calibrated cuff-based SphygmoCor device (AtCor Medical, Sydney, Australia). cfPWV was measured via carotid applanation tonometry and a femoral thigh cuff. Peripheral brachial pressure waveforms were recorded and mathematically transformed using a validated generalised transfer function to derive the central aortic waveform, from which cSBP, cDBP, PP, central augmentation pressure, and AIx corrected for a heart rate of 75 (AIx@75) were estimated.^7^ Measurement reproducibility was evaluated in 10% of participants through duplicate intra- and inter-operator assessments; readings with a cfPWV difference ≤ 0.5 m/s and peripheral systolic pressure difference ≤ 5 mmHg were accepted. Predefined cut-points classified PWV > 10 m/s as elevated, AIx@75 ≥ 40 as abnormal, cSBP ≥ 140 mm Hg and cDBP ≥ 90 mm Hg as elevated, PP > 40 mm Hg as widened, and MAP > 100 mm Hg as elevated.^8,9^ Cardiovascular risk variables including lipid profiles, HbA1c, C-reactive protein (CRP), HIV status, smoking, alcohol use, and anthropometrics were collected via standardised case report forms. Given the limited sample size, analyses were restricted to descriptive statistics with proportions and 95% confidence intervals (CIs). All statistical analyses were conducted using R statistical software (Version 4.3). Ethical statement: approval was granted by the local ethics committee (KNCHREC003) and the National Health Research Ethics Committee (NIMR/HQ/R.8a/Vol.IX/2988). Written informed consent was obtained from all participants prior to enrolment.

A total of 36 participants were enrolled in the study: 13 with TB only, 11 with DM only, and 12 with TB/DM comorbidity. Six out of ten were aged 35–50 years, and men constituted 60% of the overall group of participants. HIV co-infection was present in 23% and 27% of the TB only and TB/DM groups, respectively, and was absent in the DM only group. PWA results are presented in the Table. The highest proportion of elevated cfPWV (>10 m/s) was found among those with DM only (46%, 95% CI: 16.7%–77%). Abnormal AIx@75 (≥40%) was most prevalent (25%) in the TB/DM group. Widened PP (>40 mm Hg) was the most prevalent PWA abnormality, occurring most commonly in the DM only group (55%). Elevated MAP (>100 mm Hg) was also most prominent in the DM only group, observed in 73%. Among the traditional cardiovascular risk factors, elevated HbA1c (≥48 mmol/mol [>6.5%]) was observed in 100% and 91% of participants in the TB/DM and DM only groups, respectively. The DM only group had the highest proportion of participants with an elevated LDL:HDL ratio (≥2.5), affecting 27% of the participants. Elevated CRP (>10 mg/L) was observed across all three groups, with the highest proportion in the TB/DM group (67%).

**Table.** Pulse wave analysis results and selected cardiovascular risk variables by disease group.

Variable	TB only (n = 13), n (%, 95% CI)	DM only (n = 11), n (%, 95% CI)	TB/DM (n = 12)
Patient demographics			
Age 35–50 years	8 (61.5, 31.6–86.1)	4 (36.4, 10.9–69.2)	9 (75.0, 42.8–94.5)
Age >50 years	5 (38.5, 13.9–68.4)	7 (63.6, 35.4–84.8)	3 (25.0, 5.5–57.2)
Male sex	8 (61.5, 31.6–86.1)	7 (63.6, 30.8–89.1)	7 (58.3, 27.7–84.8)
BMI underweight (<18.5 kg/m^2^)	2 (15.4, 1.9–45.4)	0 (0)	2 (16.7, 2.1–48.4)
BMI overweight (25–29.9 kg/m^2^)	5 (38.5, 13.9–68.4)	4 (36.4, 10.9–69.2)	2 (16.7, 2.1–48.4)
BMI obese (≥30 kg/m^2^)	1 (7.7, 0.2–36.0)	1 (9.1, 0.2–41.3)	1 (8.3, 0.2–38.5)
Pulse wave analysis			
cfPWV > 10 m/s	2 (15.4, 1.9–45.4)	5 (45.5, 16.7–76.6)	2 (16.7, 2.1–48.4)
AIx@75 > 40%	1 (7.7, 0.4–37.9)	2 (18.2, 3.2–52.2)	3 (25.0, 6.7–57.2)
PP > 40 mm Hg	3 (23.1, 5.0–53.8)	6 (54.5, 23.4–83.3)	4 (33.3, 9.9–65.1)
cSBP ≥ 140 mm Hg	2 (15.4, 1.9–45.4)	3 (27.3, 6.0–61.0)	1 (8.3, 0.2–38.5)
cDBP ≥ 90 mm Hg	5 (38.5, 15.1–67.7)	4 (36.4, 12.4–68.4)	5 (41.7, 16.5–71.4)
MAP > 100 mm Hg	8 (61.5, 31.6–86.1)	8 (72.7, 39.0–94.0)	5 (41.7, 15.2–72.3)
Traditional cardiovascular risk factors			
Elevated CRP > 10 mg/L	8 (57.1, 29.6–81.1)	6 (40.0, 17.4–67.1)	6 (66.7, 30.9–91.0)
HbA1c ≥ 48 mmol/mol	1 (7.7, 0.2–36.0)	10 (90.9, 58.7–99.8)	11/11 (100%**^A^** 71.5–100)
Elevated total cholesterol > 5.17	1 (7.1, 0.37–35.8)	1 (6.7, 0.35–34.0)	1 (11.1, 0.58–49.3)
Elevated LDL:HDL ≥ 2.5	2 (13.3, 2.3–41.6)	4 (26.7, 8.9–55.2)	1 (10, 0.52–45.9)
HIV positive	3 (23.1, 5.0–53.8)	0 (0, 0–26.5)	3 (27.3, 6.0–61.0)
Current smoker	1 (7.7, 0.2–36.0)	0 (0)	1 (8.3, 0.2–38.5)

Data presented as n (%, 95% CI) unless otherwise stated.

AIx@75 = augmentation index corrected to heart rate of 75 beats/min.; BMI = body mass index; cDBP = central diastolic pressure; cfPWV = carotid-femoral pulse wave velocity; CRP = C-reactive protein; cSBP = central systolic blood pressure; DM = diabetes mellitus; HbA1c = glycated haemoglobin; HDL = high density lipoprotein; LDL = low density lipoprotein; MAP = mean arterial pressure; PP = pulse pressure; PWA = pulse wave analysis.

A HbA1c measurements were available for 11 of 12 participants in the TB/DM group.

This pilot study provides preliminary evidence of subclinical vascular alterations among individuals with TB, DM, and TB/DM comorbidity in Tanzania. Interestingly, the proportion of participants with elevated cfPWV was nearly three-fold higher in the DM only group than in the TB/DM and TB only groups. The older age distribution in the DM only group may partly explain this. Furthermore, the absence of a similar pattern in the TB/DM group may suggest that, in some individuals, hyperglycaemia reflects transient TB-induced infection-related hyperglycaemia rather than established DM.^10^

Elevated MAP was frequent among participants with TB only and DM only. Unexpectedly, this pattern was less pronounced among participants with TB/DM comorbidity, despite the anticipated additive cardiovascular effects of both conditions. This finding may reflect the relatively small sample size, or selection of healthier survivors who returned for follow-up assessment. Elevated CRP, particularly observed in TB-related groups (around 60%), may reflect ongoing systemic inflammation, which has been associated with arterial stiffness.^11^ These findings suggest the potential role for non-invasive PWA as a practical cardiovascular screening tool within TB and DM care platforms in LMICs alongside established cardiovascular risk factors and clinical assessments. PWA is reproducible and relatively a simple method for detecting subclinical vascular changes which can be integrated into routine care in identifying individuals at increased cardiovascular risk among patients with TB, DM, and TB/DM comorbidity before overt CVD develops. In TB programmes, a pragmatic approach could involve targeted PWA among patients with DM, hypertension, or other cardiovascular risk factors at baseline and during treatment follow-up.

In conclusion, markers of subclinical arterial stiffness were common across all three groups of individuals with TB, DM, and TB/DM comorbidity in this pilot study from Tanzania. Although differences in vascular abnormalities and cardiovascular risk profiles were observed between the groups, the small sample size and consequent substantial overlap in confidence intervals precluded clear conclusions regarding between-group differences. These preliminary findings support broader cardiovascular risk evaluation and call for adequately powered prospective studies in individuals with TB, DM, and TB/DM comorbidity to further investigate subclinical vascular abnormalities and their relationship with cardiovascular risk.
